# Assessing Pharmacists’ Preferences towards Efficacy Attributes of Disease-Modifying Therapies in Relapsing-Remitting Multiple Sclerosis

**DOI:** 10.3390/pharmacy8020061

**Published:** 2020-04-07

**Authors:** Iciar Martínez-López, Jorge Maurino, Patricia Sanmartín-Fenollera, Ana Ontañon-Nasarre, Alejandro Santiago-Pérez, Isabel Moya-Carmona, Carlos Gustavo García-Collado, Raquel Fernández-Del Olmo, Elena García-Arcelay, Mònica Sarmiento, Ágata Carreño, Montserrat Pérez-Encinas

**Affiliations:** 1 Department of Pharmacy, Hospital Universitari Son Espases, 07120 Palma, Balearic Islands, Spain; iciar.martinez@ssib.es; 2 Medical Department, Roche Farma S.A., 28042 Madrid, Spain; elena.garcia_arcelay.eg1@roche.com; 3 Department of Pharmacy, Hospital Universitario Fundación Alcorcón, 28922 Alcorcón, Madrid, Spain; psanmartin@fhalcorcon.es (P.S.-F.); mperez@fhalcorcon.es (M.P.-E.); 4 Department of Pharmacy, Hospital Universitario de Fuenlabrada, 28942 Fuenlabrada, Madrid, Spain; ana.ontanon@salud.madrid.org; 5 Department of Pharmacy, Hospital Universitario Infanta Leonor, 28031 Madrid, Spain; asantiagop@salud.madrid.org; 6 Department of Pharmacy, Hospital Universitario Virgen de la Victoria, 29010 Málaga, Spain; moyacarmonaisabel@yahoo.es; 7 Department of Pharmacy, Hospital Universitario Virgen de las Nieves, 18014 Granada, Spain; carlosg.garcia.sspa@juntadeandalucia.es; 8 Market Access Department, Roche Farma S.A., 28042 Madrid, Spain; raquel.fernandez@roche.com; 9 Health Economics and Outcomes Research, IQVIA Information S.A., 08025 Barcelona, Spain; msarmiento@es.imshealth.com (M.S.); acarreno@es.imshealth.com (Á.C.)

**Keywords:** multiple sclerosis, pharmacist, preferences, disease-modifying therapy, disease progression

## Abstract

**Introduction:** Hospital pharmacists are increasingly playing a critical role in the care of patients with multiple sclerosis (MS). However, little is known about their preferences and perspectives towards different attributes of disease-modifying therapies (DMTs). The objective of this research was to assess pharmacists´ preferences for DMT efficacy attributes. **Methods:** A multicenter, non-interventional, cross-sectional, web-based study was conducted. Preventing relapses, delaying disease progression, controlling radiological activity, and preserving health-related quality of life (HRQoL) and cognition were the attributes selected based on a literature review and a focus group with six hospital pharmacists. Conjoint analysis was used to determine preferences in eight hypothetical treatment scenarios, combining different levels of each attribute and ranking them from most to least preferred. **Results:** Sixty-five hospital pharmacists completed the study (mean age: 43.5 ± 7.8 years, 63.1% female, mean years of professional experience: 16.1 ± 7.4 years). Participants placed the greatest preference on delaying disease progression (35.7%) and preserving HRQoL (21.6%) and cognition (21.6%). Importance was consistent in all groups of pharmacists stratified according to demographic characteristics, experience, research background, and volume of patients seen per year. **Conclusions:** Understanding which treatment characteristics are meaningful to hospital pharmacists may help to enhance their synergistic role in the multidisciplinary management of patients with MS.

## 1. Introduction

Multiple sclerosis (MS) is a chronic inflammatory demyelinating disease of the central nervous system associated with disability progression and a high impact on patients’ daily activities and quality of life (HRQoL) [1,2]. The cause of MS is unknown. Nevertheless, several risk factors have been described, including genetic inheritance, sex, age, race, climate, infections, levels of vitamin D, and smoking [3]. Recent research suggests that there is an increased risk of breast, bladder, and brain neoplasms among MS patients. The variation of risk for certain tumors could be related to lifestyle changes, therapies, or a combination of both [4]. 

Different outcomes have been classically used in clinical practice to assess the disease progression in patients with relapsing-remitting MS: the occurrence of relapses, disability progression, cognitive impairment, and neuroimaging findings [5]. The concept of “no evidence of disease activity” (NEDA) is emerging as a new standard for treatment outcomes based on the absence of relapses, disability progression, and radiological activity [6]. Over recent years, several innovative disease-modifying treatments (DMTs) have been developed and approved to manage MS [7]. While the efficacy remains the most valuable characteristic, the choice of new agents also involves other aspects, including mechanisms of action, onset and duration of effect, tolerability and safety, administration route or frequency, and patient preferences [7,8]. Different patient-reported outcomes are available to capture perceptions of health status, functional level, and quality of life, such as the 29-item MS Impact Scale (MSIS-29) and the Modified Fatigue Impact Scale (MFIS) [9]. However, they have been inconsistently employed in MS [10].

Multidisciplinary teams, including neurologists, nurses, and pharmacists, are currently involved in the management of MS patients [11]. Understanding treatment preferences of each group can be crucial in the decision-making process. Patients´ perspectives and preferences may differ from healthcare professionals´ priorities [12,13,14,15,16]. Arroyo et al. found that safety risk minimization and route and frequency of administration were the most important treatment attributes in a sample of 221 patients with relapsing-remitting MS in Spain [17]. Reducing symptomatic impact, delaying disease progression, or reducing the relapse rate were the most preferred attributes in a recent systematic review of 22 studies assessing patient information about DMTs [18]. Safety was the most important DMT attribute in the treatment decision by neurologists and nurses in a Dutch study, followed by the efficacy on disability progression, quality of life, and relapse rate [12]. However, risk of severe adverse events was valued significantly lower by patients (*b* = −2.59, *p* < 0.001). 

In this context, hospital pharmacists are increasingly playing a critical role in the care of people with MS [16]. They are involved in drug dispensation, patient education, management of adverse reactions or follow-up support, and treatment adherence evaluation [19,20,21]. However, there are no data on their preferences for different attributes of MS treatments. The objectives of this study were to determine hospital pharmacists’ preferences for different hypothetical relapsing-remitting MS DMTs according to a set of selected attributes of efficacy and to quantify the weight of each studied attribute. 

## 2. Materials and Methods 

We conducted a multicenter, non-interventional, cross-sectional, web-based study from 24 August 2018 to 30 January 2019 (ATTRIBUTE-MS study). Practicing pharmacists actively involved in the care of patients with MS were invited to participate in our study by the Spanish Society of Hospital Pharmacy (Sociedad Española de Farmacia Hospitalaria-SEFH). Informed consent was obtained from all participants. The study was approved by the Research Ethics Board of the Hospital Universitario Clínico San Carlos (Madrid, Spain). 

Preferences elicitation is a way of assessing the value that subjects assign to the different attributes of a given product and it has been used to know the value that stakeholders assign to the different attributes of medical interventions [22,23]. One technique of preferences elicitation is conjoint analysis (CA), a multivariate method based on mathematical psychology to evaluate health-related decisions requiring consideration of risks and benefits [24]. CA has been successfully applied to assess preferences for a diverse range of health interventions in different settings [25,26].

Treatment efficacy attributes and levels were selected through a review of relapsing-remitting MS clinical trials and patient preferences literature and, finally, were confirmed in a focus group formed by six hospital pharmacists with expertise in MS [5,27]. Delaying disease progression, preventing relapses, subclinical disease activity control based on magnetic resonance imaging, preserving cognition, and preserving health-related quality of life were the five attributes selected (Table 1). Finally, an orthogonal design was implemented to develop eight hypothetical treatment scenarios containing unique combinations of attributes and levels (Table 2). Participants were asked to rank scenarios from position 1 (most preferred) to position 8 (least preferred). 

The primary analysis was focused on pharmacists´ preferences for the attributes of DMTs. The ordinary least squares (OLS) method was selected to estimate parameters. Results were summarized in terms of utilities (profits), relative (overall) and individual (at pharmacist level) importance assigned to each attribute, the Kendall correlation coefficient between real ranges and those predicted by the model were used to assess the goodness of fit of the model, and inverse preferences were evaluated (pharmacists whose preferences were the opposite of the expected relationship). The relative importance of each factor was obtained by dividing the importance of a factor by the sum of all individual importance scores.

In addition, a k-means clustering procedure based on individual profits provided by pharmacists to different attributes was used to define the profiles of pharmacists with similar preferences. Differences in sociodemographic variables were assessed between clusters. A difference analysis was performed grouped into 2, 3, and 4 clusters. The cluster assigned to each participant was saved to compare pharmacists’ characteristics and importance for each attribute to characterize each cluster.

A sensitivity analysis was performed excluding those pharmacists with inverse preferences. Individual importance of each attribute (or level) was explored according to different sociodemographic variables using bivariate tests. 

The statistical analysis was performed using SPSS version 19.0 (IBM: Armonk, NY, USA), which uses OLS algorithms to estimate profits. Also, SAS version 9.4 (SAS Institute Inc.: Cary, North Carolina 27513, USA) was used to generate some results. 

## 3. Results

A total of 65 hospital pharmacists were included in the study. The mean (SD) age was 43.5 (7.8) years and 63.1% were female. The main sociodemographic characteristics of the sample are shown in Table 3. 

### 3.1. Conjoint Analysis

All participants agreed to consider the F scenario as the first selected option, selecting the best combination as the most preferred MS treatment. Participants placed the greatest relative importance on delaying disease progression (35.7%), followed by preserving HRQoL (21.6%) and cognition (21.6%) (Table 4). Similar values were obtained with average importance: 36.3%, 21.8%, and 18.2%, respectively. Estimated utilities reported by pharmacists for attributes and levels were consistent in groups of pharmacists stratified according to demographic characteristics, experience, research background, and volume of patients seen per year.

Pearson’s R and Kendall’s τ coefficients showed a high correlation: 0.996 (*p* < 0.001) and 0.857 (*p* < 0.001), respectively. Importance assigned to the different attributes showed minor differences according to the method used (average or relative importance).

The sensitivity analysis, excluding those pharmacists who showed individual reversed utilities in cognitive impairment and relapses, assigned similar values in utilities for DMTs, increasing the relative importance of disease progression (38.4%), but slightly lowering the importance assigned to the rest of the attributes. 

**Figure 1:**
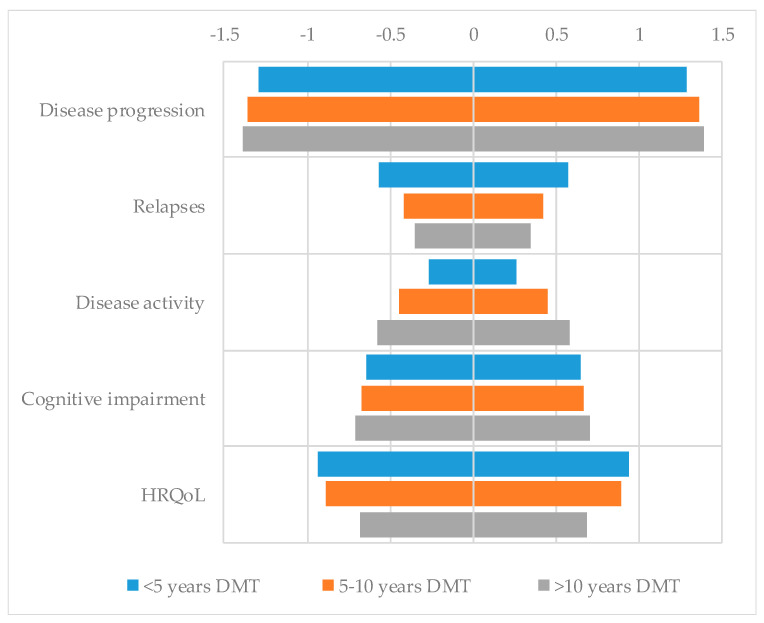
Utility scores of pharmacists for each level of each attribute according to years of experience managing disease-modifying therapies (DMTs).

An exploratory CA, according to the number of years of experience managing DMTs (less than 5 years [n = 19], between 5 and 10 years [n = 18], and more than 10 years [n = 28]), was conducted (Figure 1). Overall, no relevant differences were observed between different groups. Those pharmacists with a longer experience in DMTs seem to have slightly higher preference for treatments with better efficacy in terms of disease progression, cognition, and control of radiological activity. 

### 3.2. Cluster Analysis

An exploratory cluster analysis was performed to identify possible profiles based on preferences for attributes and levels. Participants were grouped into three clusters according to the importance assigned to each attribute:

1. CLUSTER 1: Participants clearly preferring MS treatments with low impact on the HRQoL and slightly higher importance of preventing relapses (n = 14, 21.5%).
2. CLUSTER 2: Participants clearly preferring MS treatments preserving cognition (n = 15, 23.1%).
3. CLUSTER 3: Participants clearly preferring MS treatments delaying disease progression and controlling radiological disease activity (n = 36, 55.4%).

## 4. Discussion

The main goals of therapy in relapsing-remitting MS involve a balance between control of disease activity and optimization of tolerability and safety [33,34]. Multidisciplinary teams currently face more complex treatment decisions when considering specific disease targets, including relapses, disability progression, cognition, brain and spinal cord lesions, and brain atrophy [35]. The idea of a personalized risk assessment is also crucial. Autoimmune disorders may have a higher risk of certain neoplasms and this risk may be increased by immunomodulatory therapies [36].

The impact of relapsing-remitting MS patient preferences towards DMTs has begun to be explored in the last decade. A recent systematic review of 31 articles focused on patient preferences for treatments showed that efficacy, mode and frequency of administration, and side-effect profile were the most important attributes [37]. 

Hospital pharmacists are increasingly playing an essential role in the management of MS patients by contributing to the dispensation of DMTs and counselling patients and their families about administration, dosing, and risks of different side effects [19,38]. The intervention of pharmacists has shown a relevant impact on patients´ outcomes measures, quality of life, and therapeutic adherence [20,21,39]. However, hospital pharmacists’ preferences for different attributes of relapsing-remitting MS therapies had not been previously studied.

In the present study, delaying disease progression was the most prioritized DMT attribute, followed by preserving HRQoL and cognition in a sample of pharmacists caring for patients with MS at the hospital level in Spain. Cluster analysis revealed that attributes like disease progression and disease activity were related in the sense that almost half of the sample had a greater preference for both attributes. 

Kremer et al. found that safety, effect on disability progression, and quality of life were the most impactful attributes of DMTs for healthcare professionals managing MS patients [12]. Neurologists and nurses did not differ significantly in their perspective about which attributes are the most important when making decisions [12]. The authors also included the quality of life dimension for the first time in the research of DMT attributes. These results are in line with our study, showing that pharmacists involved in MS care also consider delaying disease progression as a key treatment attribute along with quality of life and cognition.

According to The Multiple Sclerosis Trend Report, most neurologists believe that specialty pharmacists can add value to improve the care of patients with MS because of the wide variety of existing therapies and the potential influence they may have on patient adherence and education as result of their high level of involvement with patients: 42% of pharmacists had contact with a given patient once every few weeks and 37% once a month [40]. 

Understanding how different healthcare professionals value the different aspects of an intervention in health care is crucial to both the design and evaluation of therapies. Incorporating these values in decision-making may ultimately result in clinical, licensing, reimbursement, and policy decisions that better reflect the preferences of every stakeholder. However, the methods to assess DMT attributes should be improved, including greater use of qualitative research for attribute/level development, sample size considerations, and design of a stated preference survey [12,26].

Our study had several limitations. We had to select treatment attributes and levels based on previous studies conducted with patients, neurologists, and nurses because of the lack of specific research on MS DMT preferences involving pharmacists. In a focus group of six hospital pharmacists with expertise in MS, it was decided to focus only on efficacy attributes to avoid the complexity of the design with multiple attributes and the difficulty of completing the exercise of selecting preferences. There is no consensus on the number of attributes to include or the number of scenarios that should be evaluated by each respondent in conjoint analysis. Most studies usually include 6 attributes [24]. Each attribute most often includes either two or three levels. Coast et al. found a higher completion rate for the 8-scenario questionnaire (85%) than for the 16-scenario questionnaire (83%) in a randomized, controlled trial to determine the completion rate for conjoint analysis studies [41]. However, the inclusion of fewer DMT attributes could have caused omitted variable bias owing to exclusion of additional key attributes already identified by patients and other healthcare professionals in previous studies, such as mode of administration, side effects, or cost of treatments [12,37]. In addition, there may be other uncollected pharmacist characteristics that could explain differences in terms of preference for DMT options. Another limitation is the lack of analysis of different treatment frameworks (escalation versus highly effective treatment early approach) in our study [42].

## 5. Conclusions

Given the growing relevance of value-based clinical decision-making in current health systems, the important role of hospital pharmacists in managing therapeutic options in diseases with a wide range of treatments, and the lack of studies that consider their point of view, this study provides an additional and novel perspective on the management of DMTs for relapsing-remitting MS.

Understanding which treatment characteristics are meaningful to pharmacists may help to enhance their synergistic role in the multidisciplinary management of patients with MS. Further studies are necessary to gather a more comprehensive spectrum of pharmacist-relevant attributes.

## Figures and Tables

**Table 1:** Efficacy attributes and levels.

Attributes	Levels	Description
Disease progression	2	- No progression: EDSS score stability or increase of <1.0 (if prior EDSS is 0–5.5) or stability if prior EDSS is >5.5 plus motor worsening <20% in T25FW and/or 9-HPT tests in 5 years - Progression: EDSS score increase of ≥1.0 (if prior EDSS 0–5.5) or ≥0.5 (if prior EDSS > 5.5) plus motor worsening ≥20% in T25FW and/or 9-HPT tests in 5 years
Relapses	2	- No relapses in 5 years - No relapses in 3 years
Subclinical disease activity ^1^	2	- No subclinical disease activity on MRI - Subclinical disease activity on MRI
Cognition	2	- No cognitive impairment (PASAT score <35) - Cognitive impairment (PASAT score ≥35)
HRQoL	2	- Low impact on HRQoL (<53 and <27 scores on physical and psychological MSIS-29 subscales, respectively) - High impact on HRQoL (≥53 and ≥27 scores on physical and psychological MSIS-29 subscales, respectively)

^1^ Subclinical disease activity: Presence of gadolinium-enhancing T1 lesions and/or new/enlarging T2-hyperintense lesions on MRI. Abbreviations: EDSS = Expanded Disability Status Scale [28]; HRQol = Health-related quality of life; MRI = Magnetic Resonance Imaging; MSIS-29 = Multiple Sclerosis Impact Scale [29]; 9-HPT = Nine-Hole Peg Test [30]; PASAT = Paced Auditory Serial Addition Test [31]; T25FW = Timed-25 Foot Walk Test [32].

**Table 2:** Set of efficacy scenarios.

Scenarios	Disease Progression	Relapses	Subclinical Disease Activity	Cognition	HRQoL
A	Disease progression	No relapses in 3 years	Subclinical disease activity	Cognitive impairment	Low impact
B	Disease progression	No relapses in 3 years	No subclinical disease activity	No cognitive impairment	High impact
C	Disease progression	No relapses in 5 years	Subclinical disease activity	No cognitive impairment	High impact
D	No disease progression	No relapses in 5 years	Subclinical disease activity	Cognitive impairment	High impact
E	No disease progression	No relapses in 3 years	No subclinical disease activity	Cognitive impairment	High impact
F	No disease progression	No relapses in 5 years	No subclinical disease activity	No cognitive impairment	Low impact
G	No disease progression	No relapses in 3 years	Subclinical disease activity	No cognitive impairment	Low impact
H	Disease progression	No relapses in 5 years	No subclinical disease activity	Cognitive impairment	Low impact

Abbreviations: MRI = Magnetic Resonance Imaging.

**Table 3:** Main characteristics of the sample.

	N = 65
Sex, female, n (%)	41 (63.1%)
Age, years, mean (SD)	43.5 (7.8)
Years of experience as a hospital pharmacist, mean (SD)	16.1 (7.4)
Years of experience managing MS DMTs	8.4 (5.4)
Participation in MS clinical trials, n (%)	13 (20.0%)
Authorship of scientific manuscripts/abstracts in peer-reviewed journals/congresses, n (%)	51 (78.5%)
MS patients/year managed in the hospital, mean (SD)	261.7 (215.8)
Management of more than 250 MS patients/year, n (%)	26 (40.0%)

Abbreviations: DMTs = Disease-modifying therapies; MS = Multiple sclerosis; SD = Standard deviation.

**Table 4:** Utility scores and importance assigned to each attribute and level by pharmacists.

Variable	Utility Estimation (SE)	Importance (Relative)	Importance (Averaged)
**Disease progression**			
No progression in 5 years	1.350 (0.114)	35.7%	36.3%
Progression in 5 years	−1.350 (0.114)		
**Relapses**			
No relapses in 5 years	0.431 (0.114)	12.1%	11.6%
No relapses in 3 years	−0.431 (0.114)		
**Subclinical disease activity**			
No disease activity on MRI	0.450 (0.114)	11.6%	12.1%
Disease activity on MRI	−0.450 (0.114)		
**Cognition**			
No cognitive impairment	0.677 (0.114)	19.0%	18.2%
Cognitive impairment	−0.677 (0.114)		
**HRQoL**			
Low impact on HRQoL	0.812 (0.114)	21.6%	21.8%
High impact on HRQoL	−0.812 (0.114)		

Abbreviations: HRQol = Health-related quality of life; MRI = Magnetic Resonance Imaging; SE = Standard error.
